# Supplementary material for: Functional Analyses of a Rhodobium marinum RH-AZ Genome and Its Application for Promoting the Growth of Rice Under Saline Stress
Source: Plants (Basel). 2025 Aug 13;14(16):2516. doi: 10.3390/plants14162516 (PMC12389409; doi:10.3390/plants14162516)
Supplement: Supplementary file 1 [file plants-14-02516-s001.zip › Table S1 RH-AZ genomic annotation for plant growth promotion and salt stress tolerance.pdf]

Table S1. RH-AZ genomic annotation for plant growth promotion and salt stress tolerance.

Trait /Activity	Gene name	Gene ID	Gene Annotation
nitrogen fixation	fixA, fixB, fixC, fixG, fixH, fixI, fixR, fixS, fixU, nifB, nifQ, nifU, nifX, nifZ	orf00983, orf00984, orf00985, orf00397, orf00398, orf00399, orf02951, orf00400, orf00967, orf00964, orf00977, orf00979, orf00974, orf00966	Nitrogen fixation Protein FixA, FixB, FixC, FixG, FixH, FixI, FixR, FixS, FixU, NifB, NifQ, NifU, NifX, NifZ
	fixX	orf00986	Ferredoxin-like protein
	nifA	orf00963	Nif-specific regulatory protein
	nifD1	orf00970	Nitrogenase molybdenum-iron protein alpha chain
	nifE	orf00972	Nitrogenase iron-molybdenum cofactor biosynthesis protein NifE
	nifH	orf00969	Nitrogenase iron protein
	nifK1	orf00971	Nitrogenase molybdenum-iron protein beta chain
	nifN	orf00973	Nitrogenase iron-molybdenum cofactor biosynthesis protein NifN
	nifS	orf00980, orf01713	cysteine desulfurase NifS
	nifV	orf00981	Homocitrate synthase
	nifW	orf00982	Nitrogenase-stabilizing/protective protein NifW
Nitrogen metabolism	nirK	orf00008	Copper-containing nitrite reductase
	BCA2	orf01046	Beta carbonic anhydrase 2, chloroplastic
	glnA	orf01701	Glutamine synthetase
	glnB	orf01700, orf03363, orf03365	Nitrogen regulatory protein P-II
	glnD	orf03278	Bifunctional uridylyltransferase/uridylyl-removing enzyme
	glnL	orf03038	Sensory histidine kinase/phosphatase NtrB
	gltB	orf03536	Ferredoxin-dependent glutamate synthase 1
	gltD	orf03538	Glutamate synthase [NADPH] small chain
	norB, norC	orf00273, orf00272	Nitric oxide reductase subunit B, C
	ntrB	orf01501	Sensory histidine kinase/phosphatase NtrB
	ntrC	orf01500	DNA-binding transcriptional regulator NtrC

	ntrX	orf01498	Nitrogen assimilation regulatory protein NtrX
	ntrY	orf01499	Nitrogen regulation protein NtrY
auxin biosynthesis	trpA	orf03261	Tryptophan synthase alpha chain
	trpB	orf03263	Tryptophan synthase beta chain
	trpC	orf01641	Indole-3-glycerol phosphate synthase
	trpD	orf01640	Anthranilate phosphoribosyltransferase
	trpE	orf01638	Anthranilate synthase component 1
	trpF	orf03264	N-(5'-phosphoribosyl)anthranilate isomerase
	trpG	orf01639	Anthranilate synthase component 2
	trpS	orf03276	Tryptophan--tRNA ligase
Acetolactate synthase	ilvG	orf03389	Acetolactate synthase isozyme 2 large subunit
	ilvH	orf00942	Acetolactate synthase small subunit
	ilvI	orf00941	Acetolactate synthase large subunit
	ilvX	orf01208	Acetolactate synthase large subunit IlvX
siderophore transporter	btuC	orf02792, orf02467	iron ABC transporter permease, iron chelate uptake ABC transporter family permease
	entB	orf02591	Biosynthesis of siderophore group nonribosomal peptides
	fct	orf03374	TonB-dependent siderophore receptor
	fecE	orf01308	Fe(3+) dicitrate transport ATP-binding protein FecE
	fecE	orf02468	Fe(3+) dicitrate transport ATP-binding protein FecE
	hemH	orf03549	Ferrochelatase
	hmuU	orf02887	iron ABC transporter permease
	hmuV	orf02793	ABC-type cobalamin/Fe3+-siderophores transport
	NIFU4	orf03189	Fe-S cluster biogenesis protein NfuA
	viuB	orf02786	siderophore-interacting protein
	yvrB	orf01307	iron ABC transporter permease
phosphorus solubiliz	ALPI	orf02545	Intestinal-type alkaline phosphatase
	gcd	orf01509	Quinoprotein glucose dehydrogenase

ation	glcB	orf03713	Malate synthase G
	pckA	orf03234	Phosphoenolpyruvate carboxykinase (ATP)
	phoB	orf02928	Phosphate regulon transcriptional regulatory protein PhoB
	phoH	orf03184	PhoH-like protein
	phoR	orf02998	Alkaline phosphatase synthesis sensor protein PhoR
	phoU	orf02927	Phosphate-specific transport system accessory protein PhoU homolog
	ppa	orf00695	Inorganic pyrophosphatase
	ppk	orf01541	Polyphosphate kinase
	ppx2	orf02092	Exopolyphosphatase 2
	pstB	orf02926	Phosphate import ATP-binding protein PstB
	pstS	orf02923	Phosphate-binding protein PstS
	pyk	orf00282	Pyruvate kinase
oxidative stress alleviation	bcp	orf01724	thioredoxin-dependent thiol peroxidase
	gloB	orf03668	Hydroxyacylglutathione hydrolase
	gor	orf02197	Glutathione reductase
	gshB	orf03301	Glutathione synthetase
	gsiC	orf02898	Glutathione transport system permease protein GsiC
	gstA	orf01813	glutathione S-transferase family protein
	gstB	orf01515	Glutathione S-transferase GstB
	GTT2	orf02225	Glutathione S-transferase 2
	katE	orf02988	Catalase C
	katG	orf02840	Catalase-peroxidase
	kefBC	orf02699	Glutathione-regulated potassium-efflux system protein
	sodB	orf02442	Superoxide dismutase [Mn]
	sodC2	orf02672	Superoxide dismutase [Cu-Zn] 2
K(+)/Na(+) transport	ktrA, ktrB	orf01491, orf01492	Ktr system potassium uptake protein A, B
	mrpA, mrpB, mrpC, mrpD, mrpE	orf02430, orf02429, orf02428, orf02427, orf02426	Na(+)/H(+) antiporter subunit A, B, C, D, E

	nhaA, nhaA2, nhaA2, nhaP	orf02626, orf02563, orf03172, orf00954	Na(+)/H(+) antiporter NhaA, NhaA 2, NhaA 2, NhaP
	nhaP, nhaP2	orf00910, orf00176	K(+)/H(+) antiporter NhaP, NhaP2
Osmoprotectants: glycine betaine, proline, trehalose, choline	betA, betB, betC	orf03566, orf00960, orf02589	Oxygen-dependent choline dehydrogenase, Betaine aldehyde dehydrogenase, Choline-sulfatase
	mtgB	orf02313	Glycine betaine methyltransferase
	opuAA, opuAA, opuAB	orf00278, orf01453, orf00277	Glycine betaine transport ATP-binding protein OpuAA, OpuAA, OpuAB
	ousV, ousX	orf03200, orf03198	Glycine betaine/choline transport system ATP-binding protein OusV, Glycine betaine-binding periplasmic protein OusX
	proW, proX	orf03199, orf01454	Glycine betaine/proline betaine transport system permease protein ProW, Glycine betaine/proline betaine-binding periplasmic protein
	VP1723	orf01192	Glycine betaine/proline/choline transporter VP1723
	yehW, yehX, yehY, yehZ	orf02809, orf02810, orf02811, orf02812	Glycine betaine uptake system permease protein YehW, YehX, YehY, Glycine betaine-binding protein YehZ
	ldc	orf00864	Lysine/ornithine decarboxylase
	luxQ	orf02953	Autoinducer 2 sensor kinase/phosphatase LuxQ
	ordL	orf01041	Probable oxidoreductase OrdL
	patD	orf01302	Gamma-aminobutyraldehyde dehydrogenase
	pip	orf02179	Proline iminopeptidase
	proA, proB	orf00638, orf00635	Gamma-glutamyl phosphate, reductaseGlutamate 5-kinase
	PROC	orf01850	Pyrroline-5-carboxylate reductase
	Prorsd1	orf03373	Prolyl-tRNA synthetase associated domain-containing protein 1
	proS	orf01667	Proline--tRNA ligase
	sbnB	orf01914	N-((2S)-2-amino-2-carboxyethyl)-L-glutamate dehydrogenase
	speB	orf00831	Agmatinase
	stcD	orf02806	Probable N-methylproline demethylase

malE	orf02014	Trehalose/maltose-binding protein MalE
malF	orf00600, orf02015, orf03327	Trehalose/maltose transport system permease protein MalF
malG	orf03326	Trehalose/maltose transport system permease protein MalG
otsA, otsB	orf03689, orf03690	Trehalose-6-phosphate synthase, Trehalose-phosphate phosphatase
treS	orf01132, orf03210	Trehalose synthase/amylase TreS
treT	orf02989	Trehalose synthase
treY	orf03740	Maltooligosyl trehalose synthase
treZ	orf03739	Malto-oligosyltrehalose trehalohydrolase
