# Supplementary material for: Functional Analyses of a Rhodobium marinum RH-AZ Genome and Its Application for Promoting the Growth of Rice Under Saline Stress
Source: Plants (Basel). 2025 Aug 13;14(16):2516. doi: 10.3390/plants14162516 (PMC12389409; doi:10.3390/plants14162516)
Supplement: Supplementary file 1 [file plants-14-02516-s001.zip › Table S2 The main characteristics of the rice varieties used in the experiment.pdf]

Table S2. The main characteristics of the rice varieties used in the experiment.

Main characteristics	Rice 9311	Rice 3931
Cultivar	*Oryza sativa* subsp. *indica*	*Oryza sativa* subsp. *indica*
Reproductive cycle	135-144 days	≈142 days
Seedling morphology	dwarf and compact stature	optimally spaced architecture
Tillering ability	Weak	Strong
Plant height at maturity	115	115
Culms characteristics	sturdy	sturdy
Leaf characteristics	erect dark-green leaves; 17–18 total leaves per main culm	dark-green leaves and long-wide erect flag leaves with slight inward curling; 19–20 leaves per main culm
Plant architecture	superior plant architecture with optimal leaf morphology	semi-spreading morphology with coordinated tiller distribution
Lodging resistance	strong	strong
Salt tolerance	salt-sensitive	moderate salt-tolerant
