# Supplementary figures and images for: Functional Analyses of a Rhodobium marinum RH-AZ Genome and Its Application for Promoting the Growth of Rice Under Saline Stress
Source: Plants (Basel). 2025 Aug 13;14(16):2516. doi: 10.3390/plants14162516 (PMC12389409; doi:10.3390/plants14162516)

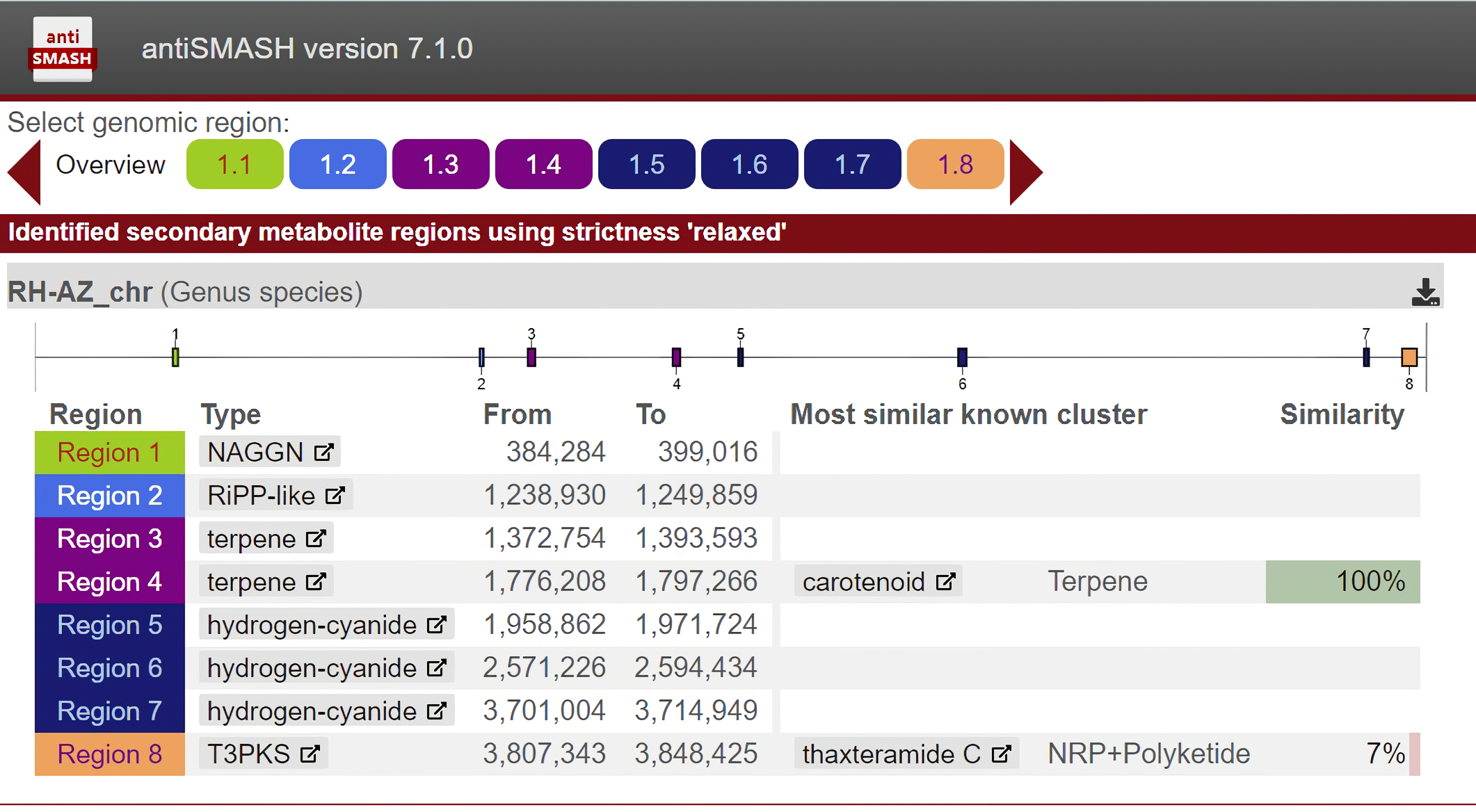

Supplement: Supplementary file 1 [file plants-14-02516-s001.zip › Figure S1 RH-AZ secondary metabolite biosynthesis was predicted via antiSMASH.png]
